# Predictor of poor coronary collaterals in chronic kidney disease population with significant coronary artery disease

**DOI:** 10.1186/1471-2369-13-98

**Published:** 2012-08-30

**Authors:** Po-Chao Hsu, Suh-Hang Juo, Ho-Ming Su, Szu-Chia Chen, Wei-chung Tsai, Wen-Ter Lai, Sheng-Hsiung Sheu, Tsung-Hsien Lin

**Affiliations:** 1 Division of Cardiology, Department of Internal Medicine, Kaohsiung Medical University Hospital, 100 Tzyou 1st Road, Kaohsiung, 80708, Taiwan, ROC; 2 Division of Nephrology, Department of Internal Medicine, Kaohsiung Medical University Hospital, 100 Tzyou 1st Road, Kaohsiung, 80708, Taiwan, ROC; 3 Department of Medical Research, Kaohsiung Medical University Hospital, 100 Tzyou 1st Road, Kaohsiung, 80708, Taiwan, ROC; 4 Faculty of Medicine, Kaohsiung, Taiwan; 5 Graduate Institute of Medicine, Kaohsiung Medical University, Kaohsiung, Taiwan; 6 Medical Genetics, Kaohsiung Medical University, Kaohsiung, Taiwan; 7 Center of Excellence for Environmental Medicine, Kaohsiung Medical University, Kaohsiung, Taiwan; 8 Department of Internal Medicine, Kaohsiung Municipal Hsiao-Kang Hospital, Kaohsiung, Taiwan

**Keywords:** Chronic kidney disease, Coronary artery disease, Coronary collateral circulation, Hypertension, Diabetes

## Abstract

**Background:**

Coronary collateral circulation plays an important role to protect myocardium from ischemia, preserve myocardial contractility and reduce cardiovascular events. Chronic kidney disease (CKD) is associated with poor coronary collateral development and cardiovascular outcome. However, limited research investigates the predictors for collateral development in the CKD population.

**Methods:**

We evaluated 970 consecutive patients undergoing coronary angiography and 202 patients with CKD, defined as a glomerular filtration rate less than 60 ml/min/1.73 m^2^, were finally analyzed. The collateral scoring system developed by Rentrop was used to classify patients into poor (grades 0 and 1) or good (grades 2 and 3) collateral group.

**Results:**

The patients with poor collateral (n = 122) had a higher incidence of hypertension (82% vs 63.8%, p = 0.005), fewer diseased vessels numbers (2.1 ± 0.9 vs 2.6 ± 0.6, p < 0.001) and a trend to be diabetic (56.6% vs. 43.8%, p = 0.085) or female sex (37.7% vs. 25.0%, p = 0.067). Multivariate analysis showed hypertension (odd ratio (OR) 2.672, p = 0.006), diabetes (OR 1.956, p = 0.039) and diseased vessels numbers (OR 0.402, p < 0.001) were significant predictors of poor coronary collaterals development. Furthermore, hypertension and diabetes have a negative synergistic effect on collateral development (p = 0.004 for interaction).

**Conclusions:**

In the CKD population hypertension and diabetes might negatively influence the coronary collaterals development.

## Background

Chronic kidney disease (CKD) is a worldwide public health problem with increased morbidity and mortality [1]. Two major poor outcomes of CKD are progression to end-stage renal disease and development of cardiovascular disease (CVD) [2]. CVD is the leading cause of morbidity and mortality in CKD patients [3]. The major risk factors in this patient group are contributed to traditional risk factors and non-traditional risk factors such as hypertension, diabetes, arterial stiffness, and so on [4,5]. Structural and functional abnormalities of heart are also another possible issues associated with excess cardiovascular risk [6,7].

The development of coronary collaterals is an adaptive response to chronic myoischemia and serves as a conduit bridging the significantly stenotic coronary vessels [8,9]. Collateral circulation can protect and preserve myocardium from episodes of ischemia, enhance residual myocardial contractility, and reduce angina symptoms and cardiovascular events [10-12]. However, there is inter-individual difference of coronary collateral formation and the mechanisms for the different individual ability to develop collateral circulation are still unclear. Chronic kidney disease is associated with poor coronary collateral vessel development in the literature [13,14]. However, there is no research discussing about the predictors of poor collaterals in the CKD population with significant coronary artery disease (SCAD). Therefore, we conducted this study for further survey.

## Methods

### Study subjects

From February 2002 to March 2008, we screened 970 consecutive patients who underwent diagnostic coronary angiography at Kaohsiung Medical University Hospital in Taiwan. Then we excluded patients with coronary artery lumen diameter stenosis <70%, estimated glomerular filtration rate (eGFR) ≧60 ml/min/1.73 m^2^, history of coronary artery bypass surgery (CABG), history of percutaneous coronary intervention (PCI), or inadequate angiograms for collateral evaluation. Finally 202 patients were analyzed in this study. CKD was defined as glomerular filtration rate <60 ml/min/1.73 m^2^. The value of eGFR was calculated using the equation in the Modification of Diet in Renal Disease study [15]. Other analyzed demographic and baseline data included gender, age, duration of chest pain, history of diabetes mellitus, hypertension, hypercholesterolemia, cigarette smoking, and medications. Duration of chest pain was defined as the time between the first occurrence of symptoms until the day of diagnostic coronary angiography. The research protocol was approved and registered by the ethics committee (Kaohsiung Medical University Hospital- Institutional Review Board) at our institution, and informed consent was obtained from all patients.

## Coronary angiography

The coronary artery angiography films were reviewed by two experienced cardiologists blind to patients’ clinical characteristics. A third reviewer blinded to the readings of the first two reviewers served as arbitrator of differences. Coronary angiography was performed by the femoral or radial approach with 6Fr diagnostic catheters. Images were recorded in multiple projections for left and right coronary arteries. Coronary artery stenosis was determined by quantitative coronary angiography. The presence of coronary artery disease (CAD) is defined as coronary diameter stenosis more than 50%. Vessels exhibiting a 70% or greater reduction in lumen area were classified as significant lesion. The recorded data also included the location, number and percentage of stenosis of diseased vessels, the vessel to which the collaterals were connected, the grade of coronary collateral circulation, and the coronary artery disease severity scoring.

### Collateral scoring and pathways evaluation by coronary angiography

In subjects with more than one SCAD vessel, the vessel with the highest collateral grade was chosen for analysis. The collateral scoring system developed by Rentrop and Cohen was used [16]. Grades of collateral filling from the contralateral vessel were: 0 = none; 1 = filling of side branches of the artery to be dilated via collateral channels without visualization of the epicardial segment; 2 = partial filling of the epicardial segment via collateral channels; 3 = complete filling of the epicardial segment of the artery being dilated via collateral channels. In subjects with more than one collateral vessel supplying the distal aspect of the diseased artery, the highest collateral grade was recorded. Patients were then classified according to their collateral grades as either poor (grade 0 or grade 1 collateral) or good (grade 2 or grade 3 collateral). In addition, the size of the collateral connection (CC) diameter was assessed by 3 grades: CC grade 0, no continuous connection between donor and recipient artery; CC grade 1, continuous, threadlike connection, and CC grade 2, continuous, small side branch-like size of the collateral throughout its course [17]. In the case of coexisting collateral connections, the prominent one was defined as the principal. The anatomic pathways were categorized according to Levin's pathways and summarized in 4 categories: septal, intra-arterial (bridging), epicardial with proximal takeoff (atrial branches), and epicardial with distal takeoff [18,19]. In the case of coexisting collateral pathways, the principal pathway was defined as the one that was the first to opacify the stenotic epicardial segments.

### Coronary artery disease severity scoring by coronary angiography

We evaluated the extent of coronary artery atherosclerosis by a “diffuse score” (DS) developed by Negri et al. and modified by Birnie et al. [20,21]. In brief, the coronary circulation is divided into 15 segments, and 8 of them are classified as first-order segments: proximal and middle right coronary arteries, left main coronary artery, proximal, middle, and distal left anterior descending, and proximal and distal circumflex. There are 7 second-order segments: distal right coronary artery, posterior descending branch, obtuse marginal branch, postero-lateral branch of circumflex, and the first 2 diagonal branches of the left anterior descending artery. The first-order segments receive a score of 1 and the second order segments scored 0.5. The overall diffuse score is the sum of the individual segment scores and the maximum score is 11.5.

### Statistical analysis

All data were expressed as means ± standard deviation. Independent t test was used to compare continuous variables between the two groups. Chi-square test was used to compare categorical data. The linear-by-linear association analysis was used to test variables interaction on the collateral development. Subsequently, significantly correlated variables in the univariate analysis or relevant variables were further analyzed by multivariate logistic regression analysis to predict the collateral development (poor vs. good). All p values were two-sided with a significance level of p < .05. The Statistical Package for the Social Sciences 11.0 for Windows (SPSS Inc.,Chicago, IL) was used for statistical analysis.

## Results

### Clinical characteristics

Among the 970 subjects initially evaluated, 768 patients were excluded for the following reasons: coronary artery lumen diameter stenosis <70%, eGFR ≧60 ml/min/1.73 m^2^, history of CABG or PCI, or inadequate angiograms for collateral evaluation. The final study population was 202 subjects (136 male and 66 female; average age, 67.2 ± 10.9 years old). For SCAD, 59 patients (29.2%) are 1VD, 62 patients (30.7%) are 2VD, and 81 patients (40.1%) are 3VD. The overall eGFR is 40.5 ± 16.3 ml/min/1.73 m^2^. 153 patients (75.7%) are CKD stage 3, 22 patients (10.9%) are CKD stage 4, and 27 patients (13.4%) are CKD stage 5.

### Coronary collaterals

The 2 collaterals readers obtained a 96% agreement in the collateral classifications. Of the 202 patients enrolled, 86 (42.6%) patients had no coronary collaterals. In subjects with collaterals, the Rentrop coronary grade was distributed as follows: 36 (17.8%) with grade 1, 60 (29.7%) with grade 2 and 20 (9.9%) with grade 3. Furthermore, we also used CC grade to provide additional information for the size of collateral connection diameter: CC grade 0 was observed in 49.5%, CC grade 1 in 37.1%, and CC grade 2 in 13.4%. For the collateral pathways: the principal pathways was through septal connections in 51.3%, atrial-epicardial connections in 26.1%, bridging connections in 13%, and distal inter-arterial connections in 9.6%. In addition, there was no significant difference between collateral grades in different CKD stages (p = 0.790).

Baseline characteristics in patients with poor and good collateral were shown in the Table 1, the patients with poor collateral (n = 122) had a higher incidence of hypertension (82% vs 63.8%, p = 0.005), and a trend to be female sex (37.7% vs. 25.0%, p = 0.067) or diabetic (56.6% vs. 43.8%, p = 0.085). Angiographic characteristics in patients with poor and good collateral were shown in the Table 2, the patients with poor collateral had a fewer diseased vessels numbers (2.1 ± 0.9 vs 2.6 ± 0.6, p < 0.001), and lower severity score of CAD (3.01 ± 1.75 vs 4.03 ± 1.83, p < 0.001).

**Table 1:** Baseline characteristics in patients with poor and good collateral

**Variables**	**Poor collateral**	**Good collateral**	**P value**
	**(n = 122)**	**(n = 80)**	
Age (years)	68.1 ± 10.1	65.7 ± 12.0	0.146
Gender (Female, %)	37.7	25.0	0.067
DM (%)	56.6	43.8	0.085
HTN (%)	82	63.8	0.005
Smoking (%)	50	42.5	0.316
BMI	25.0 ± 4.0	25.5 ± 3.6	0.351
eGFR (mL/min/1.73 m2)	40.7 ± 16.5	40.3 ± 16.0	0.853
Previous events of ACS (%)	68	58.8	0.229
**Laboratory data**			
Hemoglobin (g/dl)	12.2 ± 2.3	12.2 ± 2.6	1.000
Cholesterol (mg/dl)	198.5 ± 41.8	212.4 ± 67.8	0.104
Triglyceride (mg/dl)	187.7 ± 142.3	168.6 ± 138.1	0.360
Uric acid (mg/dl)	7.1 ± 2.2	6.9 ± 2.0	0.713
**Medication**			
Anti-platelet (%)	43.4	51.3	0.314
ACEI (%)	19.8	21.3 %	0.855
ARB (%)	21.6	24.0	0.725
Beta blocker (%)	30.8	34.7	0.636
Nitrate (%)	24.8	30.7	0.407
CCB (%)	25	17.3	0.283
Diuretic (%)	21.6	24.0	0.725
Statin (%)	18.6	27.6	0.159

ACEI: angiotensin converting enzyme inhibitor; ACS: acute coronary syndrome; ARB: angiotensin II receptor blocker; BMI: body mass index; CCB: calcium channel blocker; DM: diabetes; HTN: hypertension; eGFR: estimated glomerular filtration rate.

**Table 2:** Angiographic characteristics in patients with poor and good collateral

**Mean** **± SD**	**Poor collateral**	**Good collateral**	**P value**
**Number (%)**	**(n = 122)**	**(n = 80)**	
Number of diseased vessels	2.1 ± 0.9	2.6 ± 0.6	<0.001
Significant CAD, n (%)			
1 vessel disease (%)	47 (38.5)	12 (15.0)	
2 vessel disease (%)	41 (33.6)	21 (26.3)	<0.001
3 vessel disease (%)	34 (27.9)	47 (58.8)	
Diffuse score of CAD	3.01 ± 1.75	4.03 ± 1.83	<0.001
1 vessel disease	1.25 ± 0.67	1.42 ± 0.66	
2 vessel disease	2.72 ± 0.86	2.84 ± 0.99	
3 vessel disease	4.41 ± 1.43	4.84 ± 1.63	

CAD: coronary artery disease.

### Logistic regression analysis

Age, sex, body mass index (BMI), history of diabetes, history of hypertension, history of dyslipidemia, and number of diseased vessels were included in our univariate analysis. Age, sex, significant correlated variables and relevant variables such as history of diabetes were further analyzed by multivariate logistic regression analysis. We found diabetes (p = 0.039, OR = 1.956, 95% CI = 1.04-3.70), hypertension (p = 0.006, OR = 2.672, 95% CI = 1.32-5.40), and number of diseased vessels (p < 0.001, OR = 0.402, 95% CI = 0.26-0.62) were significant independent predictors of poor coronary collaterals development (Table 3).

**Table 3:** Multivariate logistic regression analysis of collateral circulation (good collateral group as reference group)

	**Univariate analysis**		**Multivariate analysis (forward)**	
	**OR (95% CI)**	**p value**	**OR (95% CI)**	**p value**
Age	1.020(0.99-1.05)	0.132	-	-
Sex	0.551(0.30-1.03)	0.061	-	-
Body mass index	0.965(0.90-1.04)	0.350	-	-
Diabetes	1.674(0.95-2.96)	0.076	1.956(1.04-3.70)	0.039
Hypertension	2.585 (1.35-4.95)	0.004	2.672 (1.32-5.40)	0.006
Dyslipidemia	0.945 (0.53-1.67)	0.847	-	-
Number of diseased vessels	0.472(0.32-0.70)	<0.001	0.402(0.26-0.62)	<0.001

Furthermore, diabetes and hypertension were also the most common causes of CKD, so we further analyzed the combined risk between them and found there was a synergistic effect of diabetes and hypertension on the poor coronary collateral formation (p = 0.004 for interaction; Table 4). In patients who have only diabetes or hypertension, the risk of poor collateral formation did not significantly increase (OR 1.42, 95% CI: 0.63-3.19; p = 0.395). However, when patients have both diabetes and hypertension, there is a 3.10-fold risk of poor collateral development (OR 3.10, 95% CI: 1.35-7.12; p = 0.008).

**Table 4:** Effect of diabetes and hypertension on the poor collateral development

**Patient groups**	**Poor collateral**	**Good collateral**	**OR(95% CI)**^**a**^	**P value**^**a**^
DM(−)/HTN(−)	15 (45.5%)	18 (54.5%)	1	-
DM(+)/HTN(−) or DM(−)/HTN(+)	45 (54.2%)	38 (45.8%)	1.42 (0.63-3.19)	0.395
DM(+)/HTN(+)	62 (72.1%)	24 (27.9%)	3.1 (1.35-7.12)	0.008

*HTN: hypertension; DM: diabetes; ^a^ Compared with nondiabetic and nonhypertensive group.

## Discussion

In the current study, we surveyed the predictor of poor coronary collaterals in 202 Chinese CKD patients and found there were three major findings. First, high percentages of CKD patients (42.6%) with significant coronary artery disease have no coronary collaterals. Second, hypertension and diabetes are significantly independent predictors of poor coronary collaterals. Third, there was a synergistic effect on poor coronary collateral development between hypertension and diabetes.

### CKD and poor coronary collateral formation

Tissue hypoxia is a pathologic feature of many human diseases like CAD, stroke, and kidney disease. In patients with CKD, Chronic hypoxia in the kidney has been suggested as a final common pathway to end-stage renal disease. It not only induces regulatory mechanisms but also has a significant influence on gene expression [22-24]. Several hypoxia-induced proteins such as hypoxia-inducible factor (HIF), vascular endothelial growth factor (VEGF), erythropoietin, and glucose transporter 1 are reported to have protective effects in CKD [25]. HIF and VEGF are also involved in angiogenesis and associated with coronary collateral development [26-28]. Furthermore, previous studies have reported the association between CKD and poor coronary collateral formation. Sezer M et al. stated that coronary collateral formation is significantly poorer in patients with renal failure (eGFR < 80 ml/min/1.73 m^2^) than in non-uremic (eGFR ≧ 80 ml/min/1.73 m^2^) patients [13]. Xie SL et al. also showed that lower eGFR is associated with poorer coronary vessel development in patients experiencing mild to moderate renal insufficiency [14]. Similar to previous studies, CKD patients with significant coronary artery disease have high percentage of poor coronary collaterals development. However, there is no literature discussing about the predictor of poor coronary collaterals in the CKD population with SCAD.

### The association between hypertension and coronary collaterals

Microvascular rarefaction is known as a phenomenon revealing there is smaller number of arterioles and capillaries in hypertensive subjects compared with non-hypertensive subjects [29]. Recent studies have suggested that capillary rarefaction in hypertension is likely to be a primary or a very early structural abnormality. However, the detailed mechanism is still not well understood. Impaired angiogenesis or microvascular rarefaction could contribute to increased peripheral resistance and raise blood pressure. There are several theories explaining microvascular rarefaction in hypertension. Rarefaction not only can antedate the onset of hypertension but also occurs as a consequence of prolonged elevation of blood pressure. Primary rarefaction might result from impaired angiogenesis and collateral network formation [30]. However, there are conflicting results about the association between hypertension and coronary collaterals existing in clinical studies. Few studies in the past suggest that there is a paradoxical increase in collateral circulation in patients with hypertension and CAD but Koerselman J et al. reported that hypertension is inversely associated with the presence and extent of coronary collaterals [31]. In our study, hypertension is not only correlated with poor collaterals in the univariate analysis but also a significant predictor of poor collateral formation in the multivariate analysis. These findings are similar to the study of Koerselman J et al.’s study. In addition, recent clinical research has also shown that long term treatment of hypertension can prevent microvascular rarefaction in hypertensive patients.

### The association between diabetes and coronary collaterals

Diabetic patients have a less favorable cardiovascular outcome and higher mortality than non-diabetic patients [32,33]. However, the precise mechanism is still not clearly clarified. According to the literature, high glucose levels could cause endothelial dysfunction and endothelial cell is vital for coronary collateral formation [34]. In addition, nitric oxide production, HIF-1, and vascular endothelial growth factor (VEGF) expressions, which are associated with angiogenesis, were also reported to be impaired in hyperglycemic condition.[35,36]. These findings all suggest that diabetes might lead to poor coronary collateral development. In animal studies, hyperglycemia reduces coronary collateral blood flow through a nitric oxide-mediated mechanism in dog model [37]. In human angiographic studies, Abaci A et al. demonstrated that coronary collateral development in the diabetes group was significantly poorer than in the nondiabetic group [38]. In our study, we found diabetes is a significant predictor of poor collateral formation in the multivariate analysis.

Furthermore, we also found there was a synergistic effect of hypertension and diabetes on the poor coronary collateral formation. Hypertension is both a cause and consequence of CKD. Blood pressure in CKD patients is increased due to fluid overload and production of vasoactive hormones via renin-angiotensin system which might aggravate hypertension. Diabetes is also another leading cause of CKD. Insulin and glucose homeostasis are altered in the patients of end stage renal disease and even in the early stages of CKD, leading to insulin resistance by a variety of pathways [39,40]. Insulin resistance is reported to increase with the progression of CKD, which also plays an important role in the pathogenesis of hypertension [41]. Hence, CKD might further potentiate hypertension and diabetes and cause poor coronary collateral formation.

### Limitations of the present study

First, the collateral formation was assessed by coronary angiography in this study. Measuring collateral flow index by intravascular Doppler guidewire may provide a more objective physiological measurement of collateral grade. However, the invasiveness of intravascular ultrasound limits its use in large-scale studies. Second, since this was only a clinical association study, potential mechanisms were not fully elucidated.

## Conclusions

Our data revealed that high percentages of CKD patients have no coronary collaterals. Furthermore hypertension and diabetes are both associated with poor coronary collaterals and have a negative synergistic effect on the collateral development in the CKD population. Our data might partially explain why CAD patients with CKD have poor cardiovascular outcomes. These findings could provide new insights on the risk stratification, blood pressure and glucose control for CKD CAD population.

## Competing interests

The authors declare that they have no competing interests.

## Author’s contributions

PCH, SHM, JSH, SSH, LWT, and LTH designed the study; PCH, SHM, JSH, SSH, LWT, and LTH conducted the research; CSC and TWC provided intellectual content of critical importance to the work described; PCH, and LTH analyzed and interpretated of data and wrote the manuscript. All authors read and approved the final manuscript.

## Pre-publication history

The pre-publication history for this paper can be accessed here:

http://www.biomedcentral.com/1471-2369/13/98/prepub
